# Time-restricted eating (16/8) and energy-restricted diet: effects on diet quality, body composition and biochemical parameters in healthy overweight females

**DOI:** 10.1186/s40795-023-00753-6

**Published:** 2023-08-09

**Authors:** Özge Mengi Çelik, Eda Köksal, Müjde Aktürk

**Affiliations:** 1 Faculty of Health Sciences, Department of Nutrition and Dietetics, University of Health Sciences, Ankara, Turkey; 2 Faculty of Health Sciences, Department of Nutrition and Dietetics, Gazi University, Ankara, Turkey; 3 Faculty of Medicine, Department of Endocrinology, Gazi University, Ankara, Turkey

**Keywords:** Time-restricted eating, Diet quality, Body composition, Biochemical parameters

## Abstract

**Background:**

Time-restricted eating (TRE) is a current popular dietary strategy for noncommunicable diseases. However, studies demonstrated contradictory results for it and in all dietary strategies, diet quality is an the important part of the well-being. Our study aimed to investigate the effect of TRE and energy-restricted diet (ERD) on the nutritional status and diet quality of individuals.

**Methods:**

This pilot study was completed 23 healthy overweight female. Anthropometric and body composition measurements of individuals were taken. The energy expenditure was measured using indirect calorimetry. Blood pressure and heart rate measurements were made. Biochemical parameters were evaluated and food consumption were taken. The quality of dietary intake was assessed using the Healthy Eating Index (HEI) -2015. The physical activity levels of the individuals were estimated using the physical activity record. The Statistical Package for the Social Sciences (version 22.0) software was used for all analyses. A p-value of less than 0.05 was considered to be statistically significant.

**Results:**

After 8 weeks of intervention, while no change was observed in the diet quality of the individuals in the TRE group (p > 0.05), a significant increase was found in the diet quality score of the individuals in the ERD group (p < 0.05). There was a 3.2% and 5.5% decrease in body weight of individuals in the TRE and ERD groups, respectively (p < 0.05). While no significant change was observed in the body fat percentage of individuals in the TRE group (p > 0.05), a 7.1% decrease was observed in the ERD group (p < 0.05). A statistically significant decrease was found in the total cholesterol (3.7%) in the ERD group, and in the total cholesterol (6.7%) and low density lipoprotein cholesterol (LDL-C) (6.5%) in the TRE group. In addition, a statistically significant increase was found in adiponectin (77.3%) and total antioxidant status (TAS) (13.2%) in the ERD group.

**Conclusion:**

Energy-restricted diet yielded better results in weight loss and improvement of body composition and diet quality compared to TRE. Also, a decrease in total cholesterol level was found in the ERD group. However, more studies should be done with longer follow-ups and high sample sizes are very important in terms of creating public health-based recommendations.

## Introduction

Obesity is a health problem that affects all age groups and has a high prevalence worldwide. The prevalence of obesity worldwide has nearly tripled since 1975. It has been reported that 39% of adults aged 18 years and over are overweight and 13% are obese in 2016 [1]. According to the ‘Turkey Health Survey’ data, the rate of obese individuals aged 15 and over was 19.6% in 2016, while it was 21.1% in 2019. When viewed on the basis of gender; in 2019, 24.8% of women are obese and 30.4% are overweight, while 17.3% of men are obese and 39.7% are overweight [2]. According to the World Health Organization European Regional Obesity Report 2022, while the prevalence of obesity among adults in Europe is 23.3%, 32.1% of adults in Turkey are obese and Turkey is the country with the highest obesity rate in Europe [3].

Lifestyle changes are recommended along with diets in which individual-specific energy restriction is applied to ensure body weight loss, improve body composition and metabolic health [4]. However, with this diet approach, both the goal of body weight loss is achieved in a long time and the compliance of individuals to the diet may decrease [5–7]. Intermittent fasting, which has become a popular topic recently, is an alternative energy restriction method that has emerged to provide body weight loss with dietary modifications, improve body composition, prevent or treat obesity and chronic diseases [8–11]. Time-restricted eating (TRE) is one of the most frequently used fasting protocols in the literature and includes a fasting period for various times (12–21 h/day) during the day. It is a dietary approach characterized by diurnal nutrition and prolongation of nocturnal fasting. This dietary approach provides individuals with the opportunity to be fed ad libitum without the need to calculate the energy taken outside of the fasting period. In this dietary approach, there is no intervention to change the amount of food intake of individuals [12, 13].

Time-restricted eating is a dietary approach that is based on the reduction of daily eating time and is suitable for human physiology, without night eating. Therefore, it is associated with the circadian rhythm. Unfortunately, today’s eating habits conflict with circadian physiology. The hypothesis that meal timing and increased daily eating time may cause metabolic dysfunction has been put forward in recent years. Frequent eating ensures the physiology of satiety, causes deterioration of the metabolic state during fasting, and decreases the normal circadian oscillator in metabolic organs, including the liver. It has been reported that TRE practice compatible with the circadian rhythm can have a positive effect on body composition, metabolic processes and preventing the formation of chronic diseases [13–22].

In the literature, the number of studies investigating the effect of TRE on diet quality is quite limited [23, 24]. This study was planned to investigate the effect of TRE on the nutritional status and diet quality of individuals and to compare the effects of TRE and energy-restricted diet (ERD) in healthy overweight individuals. It is thought that the study will fill an important gap in the literature.

## Materials and methods

### Data collection

This experimental study was carried out with 26 healthy overweight female individuals aged between 19 and 32 years, who applied to Gazi University Faculty of Health Sciences Nutrition and Diet Individual Counseling Center between January 2020 - May 2021. The female individuals were randomly divided into the TRE (n = 13) or ERD groups (n = 13) in an unbiased manner using a computer-generated block randomisation list. The study was completed by 23 individuals (with 10 individuals in the TRE group). Three individuals were excluded from the study because they did not adhere to the diet. Individuals who smoke, who are pregnant, lactating and who are in the postmenopausal period, who take hormone therapy, who have followed any diet program for the last 3 months before the study, and who use vitamin/mineral supplementation were not included in the study. In addition, individuals were asked to maintain their physical activity levels throughout the study. Through an application on the phone, the daily step count of the individuals was determined and their physical activity levels were followed during the study. For this reason, individuals who wanted to increase their physical activity level in addition to the applied diet were not included in the study. Consent form was signed by the individuals who accepted the study. Ethical approval was obtained from the Clinical Research Ethics Committee of Zekai Tahir Burak Women’s Health Training and Research Hospital (No 86/2019). All procedures were carried out in accordance with the Declaration of Helsinki. The data were collected using a face-to-face interview method via a questionnaire. All measurements were made on individuals in both groups.

### Diet groups involved in the study

Individuals in the TRE group applied a diet limited to 8 h for 8 weeks (16/8). They were fed ad libitum between 10.00 a.m-06.00 p.m and fasted between 06.00 p.m-10.00 a.m. No restrictions were imposed on the type and amount of food consumed during the feeding period. During the fasting period, individuals consumed water and non-energy drinks (tea, coffee, soda, etc.). Individuals’ daily caffeine intake is limited to 300 mg (about 3–4 cups of coffee or 5–6 large cups of tea).

Individuals in the ERD group followed a diet specially prepared for them for 8 weeks. At the first encounter with individuals, resting energy expenditure (REE) was measured by the indirect calorimetry method. Physical Activity Level (PAL) was determined by physical activity record. The total energy expenditure (TEE) of individuals was determined using the ‘REE x PAL’ formulation. ‘TEE-500 kcal’ formulation was used for the diet to be given to individuals [25]. Individuals were not given a diet containing energy below the REE. Acceptable macronutrient distribution range were taken as dietary content. The diet given to individuals has 45–65% carbohydrate, 20–35% fat, 10–35% protein content [26]. Nutritional habits of individuals were taken into consideration while planning the diet. Each individual consumed 3 main meals and the number of snacks was determined individually. Food exchange lists were given to individuals and nutrient changes were explained in detail. Individuals’ daily caffeine intake is limited to 300 mg (about 3–4 cups of coffee or 5–6 large cups of tea).

### Anthropometric measurements and body composition

Anthropometric and body composition measurements of individuals were taken by the researcher at the beginning and end of the study in accordance with technique. Body weight and body composition measurements were taken using an Inbody 720 body analyzer. Inbody 720 is multifrequency and has a bioelectrical impedance analysis system. The height of the individuals was measured with a stadiometer with a sensitivity of 0.1 cm. Body mass index (BMI) was calculated by dividing the body weight by the square of the height (kg/m^2^) [27]. For waist circumference measurement, the midpoint between the lowest rib bone and the cristailiac was found, and the circumference measurement passing through this point was taken. The waist/height ratio was calculated from the waist circumference and height measurements taken [28]. Neck circumference was measured with a non-flexible tape measure perpendicular to the neck axis, just below the cricoid cartilage, with the head in an upright position [29]. The degree of visceral adiposity (range 1–59 arbitrary units) and abdominal adiposity were measured with the Tanita Viscan AB 101 abdominal analyzer. Tanita Viscan AB 101 has bioelectrical impedance analysis and the amount of visceral and abdominal fat is estimated by measuring the voltage produced in this location [30].

### Determination of resting energy expenditure

Resting energy expenditure was measured at the beginning and end of the study using indirect calorimetry (COSMED, FitMatePro, Rome, Italy). Measurements were taken in the early morning hours after at least 8 h of fasting. The device is automatically calibrated before each measurement. A sterile mask covering the mouth and nose was used to determine the oxygen consumption (VO2; ml/dk). Measurements were taken in a quiet environment with a room temperature of 22–24 °C while the subjects were resting in a still and supine position. Each measurement took an average of 15 min [31, 32].

### Biochemical parameters and blood pressure

Blood samples from individuals were taken at the beginning and end of the study, after at least 8 h of fasting, in the morning hours by the Gazi University Medical Faculty Hospital blood collection unit in 10 ml yellow capped tubes and delivered to the researcher. The blood samples, which were kept for half an hour by the researcher, were then centrifuged at 3000 rpm for 15 min to ensure separation of serum and stored as samples at -80 ºC. Total cholesterol, high density lipoprotein cholesterol (HDL-C), triglyceride, fasting glucose, insulin, C-reactive protein and interleukin 6 (IL-6) were analyzed in Gazi University Medical Faculty Hospital Biochemistry Laboratory. Total cholesterol, HDL-C, triglyceride and fasting glucose analysis were performed on Beckman Coulter AU 5800 biochemistry analyzer. The calculated LDL-C level was taken from the system. Insulin analysis was performed on a Beckman Coulter DxI 800 hormone autoanalyzer operating with the chemiluminescence method. C-reactive protein analysis was measured on a Beckman Coulter Immage 800 autoanalyzer according to the nephelometric method. IL-6 analysis was performed on Roche’s Cobas e 601 hormone autoanalyzer using the electrochemiluminescence method. Homeostatic Model Assessment of Insulin Resistance (HOMA-IR) was calculated by the researcher. The HOMA-IR cut-off point was taken as 2.5 [33].

$$\begin{array}{c}{\rm{HOMA - IR = }}\left[ {\left[ {{\rm{Glucose}}\,\left( {{\rm{fasting}}} \right)\,\left( {{\rm{mmol/L}}} \right)\,} \right.} \right.\\\left. {\left. {{\rm{ \times }}\,{\rm{insulin}}\,\left( {{\rm{fasting}}} \right)\,\left( {{\rm{U/mL}}} \right)} \right]{\rm{/22}}{\rm{.5}}} \right]\end{array}$$

Leptin and adiponectin analysis were performed in a private laboratory with Sunred ELISA kits [35]. Total antioxidant status (TAS) and total oxidant status (TOS) analysis were performed on Mindray BS300 autoanalyzer with Relassay ELISA kits. Oxidative stress index (OSI) was obtained by multiplying the ratio of total oxidant status to total antioxidant status by 100. All units have been converted to µmol/L during the calculation [36].

$$\begin{array}{c}{\rm{OSI = }}\,\left( {{\rm{Total}}\,{\rm{oxidant}}\,{\rm{status}}\,{\rm{\mu mol/L}}} \right.\,\\\left. {{\rm{/}}\,{\rm{Total}}\,{\rm{antioxidant}}\,{\rm{status}}\,{\rm{\mu mol/L}}} \right){\rm{*100}}\end{array}$$

Individuals’ systolic and diastolic blood pressures and heart rate measurements were made with the automatic blood pressure monitor Omron (HEM-7121) at the beginning and end of the study. The measurement was made 3 times on the left arm after 20 min of rest and the average values were taken.

### Dietary consumption and diet quality

At the beginning of the study, it was explained how the dietary records would be taken by the expert dietitian researcher and a dietary record form was given. The amounts of consumed foods or meals to be written on the dietary record form were explained to the individuals by using the “Food and Nutrition Photo Catalogue” [37]. Dietary records of the individuals were taken by the researcher for 3 days (2 days on weekdays, 1 day on weekends) before the study, in the first and last week of the study. The energy and nutrient intakes of the individuals during the study were determined by calculating the average of the dietary records taken in the first and last week. Energy and nutrient intakes of individuals were calculated using the Nutrition Information System (BEBIS 8.0) package program. This program is a software program used in Turkey to calculate the nutritional value of foods [38]. Diet quality was assessed with the Healthy Eating Index-2015 (HEI-2015). The index consists of 13 components. HEI components are reverse scored based on their consumption of fatty acids, sodium, added sugars, and saturated fats. The total score is obtained by summing the scores of 13 components. The highest score on the index is 100 and the lowest score is zero. If the HEI-2015 total score is ≤ 50, it is classified as “poor diet quality”, 51–80 as “diet quality to be improved” and > 80 as “good diet quality” [39]. In the study, only the amount of sodium that individuals take with food was questioned, and table salt was not included in the amount of sodium.

### Physical activity

The physical activity levels of the individuals were determined at the beginning of the study using the physical activity record. The type and duration of the activities performed by the individuals were recorded in the form. The activity durations (min) were multiplied by the energy costs (Physical Activity Ratio-PAR) values according to the types of physical activities, and the values found were summed. PAL was calculated by dividing the total (sum of PAR x min values) by 1440 min. Individuals were asked to maintain their physical activity levels throughout the study. Individuals according to their physical activity levels; grouped as inactive (PAL: < 1.40), mildly active (PAL: 1.40–1.69), active (PAL: 1.70–1.99) [28]. The daily step count of individuals during the study was determined through an application on the smartphone (Samsung Health). Individuals with a daily step count of 10.000 or more were considered active [40, 41].

### Statistical analyses

The G*Power software (Version 3.1.9.6) was used to analyze the sample’s size. The sample size of a study in which the effects of TRE and ERD were primarily compared on body weight was calculated 9, with 80% power, 0.05 α error coefficient, and 1.5 effect size (Cohen’s d) for per group.

The Statistical Package for the Social Sciences (version 22.0) software was used for all analyses. Data were evaluated with descriptive statistics such as mean, standard deviation, median, number, percentage and quartile. Distribution analysis of the data was performed using the histogram, coefficient of variation ratio, Skewness, Kurtosis and Kolmogorov-Smirnov tests. Mann Whitney U test was used in independent groups and Wilcoxon test was used in dependent groups for comparison of paired groups. Differences in mean values between groups were evaluated with the Independent t test. The McNemar-Bowker test was used in dependent groups to compare categorical variables. Chi-square analysis was used to compare qualitative data and detect differences between groups. A p-value of less than 0.05 was considered to be statistically significant.

## Results

Twenty-three healthy and overweight female individuals aged between 19 and 32 years participated in the study. The characteristics of the individuals at the beginning of the study are given in Table 1. Age and BMI were similar between groups. Except for adiponectin, there was no significant difference between the groups (p > 0.05). All of the individuals were physically inactive according to their PAL values, and it was determined that there was no statistical difference in PAL values between the two groups (p > 0.05).

**Table 1:** Characteristics of individuals at the beginning of the study

Variables	TRE group (n = 10)	ERD group (n = 13)	p-value
Age (years) ($\tilde x + SD$)	24.9 ± 2.42	25.6 ± 3.79	0.109^a^
Body Mass Index (kg/m^2^) ($\tilde x + SD$)	26.9 ± 1.61	26.9 ± 1.52	0.610^a^
Fasting glucose (mg/dL)	89.00 (13.75)	91.00 (5.50)	0.901
Insulin (µIU/mL)	6.45 (4.28)	8.20 (7.10)	0.153
HOMA-IR	1.37 (1.22)	1.68 (1.70)	0.292
Total cholesterol (mg/dL)	186.50 (17.00)	191.00 (54.00)	0.804
LDL-C (mg/dL)	116.50 (17.50)	126.00 (44.00)	0.620
HDL-C (mg/dL)	54.00 (21.00)	50.00 (15.00)	0.144
Triglyceride (mg/dL)	89.00 (40.50)	82.00 (52.00)	0.780
C-Reactive Protein (mg/L)	3.98 (4.18)	1.96 (3.26)	0.154
IL-6 (pg/mL)	2.07 (1.82)	1.97 (2.92)	0.749
Total Antioxidant Status (mmol/L)	1.19 (0.23)	1.27 (0.29)	0.926
Total Oxidant Status (µmol/L)	4.38 (1.56)	4.51 (1.89)	0.756
Oxidative Stress Index	0.39 (0.12)	0.36 (0.17)	0.951
Leptin (pg/mL)	13600.26 (13338.46)	6676.70 (7111.13)	0.226
Adiponectin (ng/mL)	9377.15 (4054.99)	5482.65 (3063.65)	0.005*
Systolic Blood Pressure (mmHg)	105.00 (5.25)	104.00 (22.00)	0.641
Diastolic Blood Pressure (mmHg)	70.00 (16.75)	63.00 (20.50)	0.062
Heart rate (bpm)	79.00 (9.00)	71.00 (19.00)	0.099
Resting energy expenditure (kcal/day)	1562.50 (357.75)	1515.00 (237.00)	0.780
Total energy expenditure (kcal/day)	1992.87 (524.44)	1955.00 (294.90)	1.000
Calculated Diet (kcal/day)	-	1508.50 (139.34)	-
Physical activity level (PAL)	1.25 (0.06)	1.27 (0.03)	0.188
Number of steps during study (number/day)	6266.0 (4294.50)	6453.0 (4192.50)	0.722

Median with interquartile range, ^a^ Student t test, other tests Mann-Withney U test, *p < 0.05

HOMA-IR: Homeostatic Model Assessment of Insulin Resistance, LDL-C: Low density lipoprotein cholesterol, HDL-C: High density lipoprotein cholesterol, IL-6: Interleukin 6

The changes in anthropometric measurement, body composition and energy expenditure of individuals as a result of the eight-week diet intervention are given in Table 2. It was determined that in TRE group there was a decrease of 2.3 ± 1.84 kg (%3.2 ± 2.64). In the ERD group there was a decrease of 4.1 ± 1.41 kg (%5.5 ± 2.04) in body weight. In addition, a significant decrease was found in the waist circumference, waist/height ratio, BMI, fat free mass, fat mass, skeletal muscle mass, total body water, visceral adiposity and abdominal obesity after the intervention in the TRE group (p < 0.05). Also, a significant decrease was found in the waist circumference, waist/height ratio, BMI, fat mass, body fat percentage, total body water, visceral adiposity and abdominal obesity after the intervention in the ERD group (p < 0.05). It was determined that there was a significant increase in fat free mass percentage and body water percentage in this group (p < 0.05). The decrease in body weight, waist circumference, BMI, fat mass and body fat percentage was higher in the ERD group. Also, the increase in fat free mass percentage and body water percentage was higher in the ERD group. (p < 0.05).

**Table 2:** Changes in anthropometric measurements, body composition and energy expenditure of individuals

Variables	TRE group (n = 10)			p-value	ERD group (n = 13)				Statistical analysis between difference^a^
	At the beginning	At the end	Difference		At the beginning	At the end	Difference	p-value	
Body weight (kg)	70.20 (16.80)	69.10 (15.93)	-2.3 ± 1.84 (%3.2 ± 2.64)	0.014*	74.40 (12.45)	71.60 (11.65)	-4.1 ± 1.41 (%5.5 ± 2.04)	0.001*	0.016*
Neck Circumference (cm)	31.50 (3.00)	31.50 (3.00)	-	1.000	32.00 (0.25)	32.00 (0.50)	-0.1 ± 0.29 (%0.4 ± 0.96)	0.180	0.205
Waist Circumference (cm)	88.00 (12.00)	85.50 (12.38)	-1.2 ± 1.15 (%1.3 ± 1.33)	0.010*	88.50 (10.25)	87.00 (10.50)	-2.0 ± 1.20 (%2.3 ± 1.42)	0.001*	0.049*
Waist to Height Ratio	0.54 (0.04)	0.54 (0.05)	-0.0 ± 0.01 (%1.3 ± 1.33)	0.012*	0.53 (0.06)	0.52 (0.07)	-0.0 ± 0.01 (%2.3 ± 1.42)	0.001*	0.058
Body Mass Index (kg/m^2^)	26.25 (2.38)	25.65 (3.73)	-0.8 ± 0.69 (%3.1 ± 2.64)	0.016*	26.70 (2.60)	24.70 (3.50)	-1.5 ± 0.54 (%5.5 ± 2.05)	0.001*	0.008*
Fat Free Mass (kg)	42.50 (7.30)	41.35 (7.85)	-0.8 ± 0.74 (%1.9 ± 1.80)	0.016*	45.80 (6.75)	45.50 (5.40)	-0.6 ± 1.09 (%1.3 ± 2.28)	0.087	0.534
Body Fat Mass (kg)	28.20 (7.63)	27.35 (7.80)	-1.5 ± 1.80 (%5.0 ± 6.77)	0.037*	28.30 ± 6.70	25.00 (6.95)	-3.4 ± 0.95 (%12.3 ± 4.69)	0.001*	0.007*
Skeletal Muscle Mass (kg)	23.00 (4.28)	22.55 (4.50)	-0.4 ± 0.44 (%1.9 ± 1.99)	0.028*	25.50 (4.05)	25.00 (3.15)	-0.4 ± 0.64 (%1.4 ± 2.47)	0.080	0.709
Body Fat (%)	40.40 (2.75)	39.90 (2.15)	-0.9 ± 1.76 (%2.1 ± 4.58)	0.169	37.30 (5.85)	35.20 (5.50)	-2.7 ± 1.29 (%7.1 ± 3.68)	0.001*	0.014*
Fat Free Mass (%)	59.59 (2.79)	60.04 (2.21)	0.8 ± 1.76 (%1.35 ± 2.89)	0.169	62.66 (5.84)	65.13 (5.47)	2.8 ± 1.27 (%4.5 ± 1.99)	0.001*	0.008*
Total Body Water (kg)	31.15 (5.33)	30.30 (5.65)	-0.6 ± 0.59 (%1.9 ± 1.92)	0.028*	33.40 (5.00)	33.20 (3.95)	-0.5 ± 0.71 (%1.5 ± 2.07)	0.027*	0.732
Total Body Water (%)	43.54 (2.23)	44.00 (1.63)	0.6 ± 1.36 (%1.4 ± 3.05)	0.169	45.77 (4.45)	47.41 (4.08)	1.9 ± 0.93 (%4.3 ± 1.98)	0.001*	0.018*
Visceral adiposity	10.00 (2.50)	9.25 (2.00)	-0.8 ± 0.67 (%7.3 ± 6.85)	0.010*	9.50 (4.00)	8.50 (3.75)	-0.9 ± 0.67 (%11.2 ± 8.5)	0.005*	0.544
Abdominal adiposity (%)	43.45 (4.63)	41.70 (5.68)	-1.7 ± 1.38 (%4.0 ± 3.33)	0.005*	41.40 (7.80)	39.50 (8.40)	-2.6 ± 1.72 (%7.1 ± 5.34)	0.001*	0.154
Resting energy expenditure (kcal/day)	1562.50 (357.75)	1554.50 (278.00)	-53.1 ± 149.91	0.386	1515.00 (237.00)	1443.00 (258.50)	-103.5 ± 195.02	0.087	0.620
Total energy expenditure (kcal/day)	1992.87 (524.44)	1977.70 (390.31)	-62.5 ± 184.90	0.386	1955.00 (294.90)	1812.50 (355.70)	-132.1 ± 249.48	0.083	0.628

Median with interquartile range, ^a^ Mann-Withney U test, other tests Wilcoxon test, *p < 0.05

Changes in biochemical parameters and blood pressure of individuals are given in Table 3. After the 8-week intervention, there was a statistically significant decrease in total cholesterol and LDL-C in the TRE group (p < 0.05). It was determined that there was a significant decrease in total cholesterol and a significant increase in TAS and adiponectin in the ERD group (p < 0.05). The increase in adiponectin level was higher in the ERD group (p < 0.05).

**Table 3:** Changes in biochemical parameters and blood pressure of individuals

Variables	TRE group (n = 10)				ERD group (n = 13)				Statistical analysis between difference^a^
	At the beginning	At the end	Difference	p-value	At the beginning	At the end	Difference	p-value	
Fasting glucose (mg/dL)	89.00 (13.75)	91.50 (19.25)	-1.9 ± 8.08 (%2.1 ± 9.45)	0.386	91.00 (5.50)	85.00 (9.50)	-3.7 ± 6.22 (%3.9 ± 7.19)	0.054	0.663
Insulin (µIU/mL)	6.45 (4.28)	5.60 (6.10)	-0.2 ± 4.08 (%10.6 ± 6.74)	0.838	8.20 (7.10)	6.70 (8.65)	-1.3 ± 6.03 (%4.5 ± 71.4)	0.249	0.402
HOMA-IR	1.37 (1.22)	1.28 (1.34)	-0.1 ± 0.93 (%9.8 ± 63.73)	0.959	1.68 (1.70)	1.31 (1.73)	-0.3 ± 1.35 (%7.5 ± 72.9)	0.173	0.385
Total cholesterol (mg/dL)	186.50 (17.00)	178.00 (13.75)	-13.9 ± 12.82 (%6.7 ± 5.89)	0.008*	191.00 (54.00)	179.00 (49.00)	-6.8 ± 11.11 (%3.7 ± 6.7)	0.043*	0.145
LDL-C (mg/dL)	116.50 (17.50)	107.00 (16.25)	-9.1 ± 11.25 (%6.5 ± 9.91)	0.008*	126.00 (44.00)	117.00 (53.50)	-4.5 ± 10.75 (%3.7 ± 10.1)	0.239	0.251
HDL-C (mg/dL)	54.00 (21.00)	52.00 (11.50)	-7.7 ± 10.61 (%10.8 ± 15.35)	0.074	50.00 (15.00)	44.00 (12.00)	-3.3 ± 5.29 (%5.2 ± 10.2)	0.058	0.437
Triglyceride (mg/dL)	89.00 (40.50)	108.50 (32.75)	15.4 ± 27.41 (%24.1 ± 34.89)	0.114	82.00 (52.00)	94.00 (41.50)	4.1 ± 39.67 (%7.9 ± 39.4)	0.944	0.128
C-Reactive Protein (mg/L)	3.98 (4.18)	3.09 (3.26)	-0.8 ± 1.97 (1.8 ± 65.85)	0.241	1.96 (3.26)	1.94 (2.55)	-0.6 ± 2.85 (%4.0 ± 38.2)	1.000	0.264
IL-6 (pg/mL)	2.07 (1.82)	1.50 (0.97)	1.7 ± 7.11 (%72.0 ± 235.1)	0.866	1.97 (2.92)	2.22 (2.44)	-0.2 ± 5.57 (%66.7 ± 232.9)	0.374	0.801
Total Antioxidant Status (mmol/L)	1.19 (0.23)	1.30 (0.59)	0.04 ± 0.38 (%5.4 ± 31.58)	0.646	1.27 (0.29)	1.46 (0.36)	0.2 ± 0.20 (%13.2 ± 17.9)	0.023*	0.368
Total Oxidant Status (µmol/L)	4.38 (1.56)	5.16 (1.51)	0.23 ± 1.26 (%8.9 ± 29.27)	0.594	4.51 (1.89)	5.42 (2.92)	1.2 ± 2.36 (%33.1 ± 54.9)	0.173	0.264
Oxidative Stress Index	0.39 (0.12)	0.41 (0.11)	0.02 ± 0.09 (%7.1 ± 24.93)	0.415	0.36 (0.17)	0.44 (0.12)	0.03 ± 0.14 (%17.0 ± 43.3)	0.382	0.664
Leptin (pg/mL)	13600.26 (13338.46)	7400.58 (6655.05)	-3226.8 ± 7657.80 (%1.3 ± 78.46)	0.333	6676.70 (7111.13)	7206.72 (8755.19)	306.2 ± 4628.46 (%2.8 ± 35.8)	0.650	0.336
Adiponectin (ng/mL)	9377.15 (4054.99)	11058.93 (3393.05)	508.8 ± 2649.30 (%11.2 ± 39.82)	0.878	5482.65 (3063.65)	9393.08 (5105.05)	3189.3 ± 2760.60 (%77.3 ± 58.9)	0.006*	0.035*
Systolic Blood Pressure (mmHg)	105.00 (5.25)	108.00 (16.00)	1.6 ± 8.01 (%1.9 ± 7.91)	0.609	104.00 (22.00)	91.00 (16.00)	-3.7 ± 10.76 (%2.7 ± 11.6)	0.248	0.162
Diastolic Blood Pressure (mmHg)	70.00 (16.75)	72.50 (15.75)	1.2 ± 11.05 (%2.7 ± 16.05)	1.000	63.00 (20.50)	63.00 (11.00)	-1.4 ± 8.58 (%0.7 ± 14.1)	p = 0.529	0.686
Heart rate (bpm)	79.00 (9.00)	78.00 (15.75)	-2.0 ± 8.65 (%2.5 ± 11.12)	0.540	71.00 (19.00)	76.00 (19.50)	-0.7 ± 7.10 (%1.0 ± 10.5)	p = 0.893	0.514

Median with interquartile range, ^a^ Mann-Withney U test, other tests Wilcoxon test, *p < 0.05

HOMA-IR: Homeostatic Model Assessment of Insulin Resistance, LDL-C: Low density lipoprotein cholesterol, HDL-C: High density lipoprotein cholesterol, IL-6: Interleukin 6

The amount of energy and nutrients that individuals take in the diet are given in Table 4. In the TRE group daily energy, protein, animal protein, fat, omega-6, vitamin E, vitamin B_2_, vitamin B_6_, folate, vitamin C, potassium, phosphorus and zinc intakes were found to be significantly decreased (p < 0.05). In the ERD group individuals’ daily energy, protein, plant protein, animal protein, fat, fat percentage, carbohydrate, saturated fatty acid, polyunsaturated fatty acid, cholesterol, omega-3, omega-6, fiber, vitamin E (mg), vitamin B_1_, niacin, vitamin B_6_, sodium, potassium, magnesium, phosphorus, iron and zinc intakes were found to be significantly decreased (p < 0.05). It was determined that there was a statistically significant increase in protein percentage (p < 0.05). When the nutrient intakes between the two groups are evaluated; while energy, fat, omega-3, omega-6 and vitamin E (mg) intakes were statistically higher in the TRE group; it was determined that protein percentage, vitamin C and potassium intakes were statistically significantly higher in the ERD group (p < 0.05).

**Table 4:** Dietary energy value and nutrient intake before and after the intervention

Variables	TRE group (n = 10)		Difference	p-value	ERD group (n = 13)		Difference	p-value	Statistical analysis of during the study between the two groups^a^
	Before study	During study			Before study	During study			
Energy (kcal)	1600.67 (802.03)	1335.61 (315.91)	-405.7 ± 448.60 (%18.5 ± 19.55)	0.028*	2080.37 (678.34)	1142.64 (300.25)	-954.2 ± 578.75 (%42.2 ± 12.49)	0.001*	0.011*
Protein (g)	55.76 (27.48)	55.19 (10.22)	-12.9 ± 14.59	0.037*	76.50 (37.44)	49.72 (19.51)	-25.0 ± 21.74	0.004*	0.535
Protein (%)	15.50 (1.50)	16.00 (2.25)	0.5 ± 1.84	0.383	14.00 (3.00)	17.00 (2.50)	2.9 ± 3.92	0.031*	0.034*
Plant protein (g)	20.24 (16.10)	20.86 (4.15)	-2.4 ± 12.77	0.508	30.18 (18.08)	16.51 (7.47)	-13.2 ± 12.68	0.002*	0.055
Animal protein (g)	41.78 (10.73)	33.70 (9.34)	-10.5 ± 7.16	0.007*	40.08 (18.30)	33.32 (7.84)	-11.7 ± 13.27	0.006*	0.804
Fat (g)	88.39 (35.74)	57.74 (15.80)	-23.4 ± 23.97	0.009*	89.61 (32.36)	46.03 (13.91)	-51.8 ± 31.88	0.001*	0.005*
Fat (%)	38.50 (10.25)	38.50 (4.13)	-2.5 ± 3.74	0.092	40.00 (7.00)	35.00 (10.25)	-5.0 ± 6.00	0.019*	0.263
Carbohydrate (g)	172.483 (97.22)	160.83 (64.95)	-35.3 ± 50.54	0.093	246.98 (89.11)	126.22 (26.49)	-97.1 ± 65.58	0.001*	0.215
Carbohydrate (%)	45.50 (8.50)	45.25 (6.38)	2.2 ± 3.48	0.083	44.00 (7.00)	47.00 (10.25)	2.0 ± 7.37	0.556	0.709
Saturated fat (%)	14.04 (2.88)	12.41 (1.71)	-1.0 ± 2.46	0.203	14.40 (3.05)	11.94 (2.92)	-2.1 ± 3.58	0.046*	0.457
Monounsaturated fatty acid (%)	12.34 (5.70)	12.70 (2.94)	-0.1 ± 2.62	0.959	12.67 (3.44)	13.53 (4.64)	0.01 ± 2.47	0.972	0.901
Polyunsaturated fatty acid (%)	9.80 (4.04)	9.47 (2.75)	-1.1 ± 1.92	0.114	10.58 (5.14)	8.17 (2.54)	-2.5 ± 3.34	0.028*	0.215
Cholesterol (mg)	221.25 (114.88)	191.13 (36.12)	-53.5 ± 112.99	0.285	222.46 (173.47)	183.26 (68.16)	-87.5 ± 134.02	0.028*	0.352
Omega-3 (g)	1.57 (1.49)	1.19 (0.87)	-0.7 ± 1.59	0.169	1.21 (1.97)	0.74 (0.36)	-1.2 ± 1.80	0.016*	0.018*
Omega-6 (g)	18.48 (11.96)	13.46 (4.20)	-5.8 ± 6.80	0.013*	20.90 (9.95)	9.71 (3.06)	-11.7 ± 7.19	0.001*	0.005*
Fiber (g)	16.78 (10.95)	14.80 (3.24)	-3.3 ± 5.83	0.114	20.87 (10.78)	15.34 (5.67)	-4.2 ± 6.58	0.039*	0.620
Vitamin A (mcg)	564.52 (221.00)	663.37 (580.11)	325.5 ± 755.88	0.333	735.25 (728.51)	750.27 (494.07)	48.5 ± 644.28	0.972	0.577
Vitamin E (mg)	23.18 (12.82)	14.87 (5.30)	-7.0 ± 5.82	0.013*	22.64 (5.36)	11.97 (3.85)	-10.1 ± 3.93	0.001*	0.035*
Vitamin B_1_ (mg)	0.75 (0.49)	0.65 (0.18)	-0.1 ± 0.31	0.173	0.95 (0.46)	0.65 (0.29)	-0.2 ± 0.33	0.010*	0.926
Vitamin B_2_ (mg)	1.09 (0.21)	0.89 (0.18)	-0.1 ± 0.22	0.047*	1.23 (0.57)	1.14 (0.43)	-0.2 ± 0.43	0.328	0.172
Vitamin B_3_ (mg)	13.21 (9.29)	11.26 (3.47)	-3.1 ± 5.28	0.093	16.61 (8.99)	10.01 (3.74)	-5.5 ± 5.91	0.016*	0.321
Vitamin B_6_ (mg)	1.27 (0.63)	0.97 (0.26)	-0.4 ± 0.43	0.013*	1.47 (0.75)	1.00 (0.38)	-0.3 ± 0.43	0.023*	0.457
Vitamin B_12_ (mcg)	4.61 (1.64)	3.73 (2.31)	-0.03 ± 3.14	0.445	5.38 (2.21)	3.49 (1.57)	-1.2 ± 2.38	0.101	0.951
Folate (mg)	228.78 (39.38)	201.27 (31.85)	-26.9 ± 34.20	0.037*	232.80 (186.21)	208.33 (73.49)	-37.5 ± 104.06	0.221	0.352
Vitamin C (mg)	69.72 (97.95)	53.24 (17.46)	-40.8 ± 49.68	0.037*	57.96 (75.18)	76.37 (39.19)	1.7 ± 50.38	0.807	0.047*
Calcium (mg)	599.53 (273.46)	529.10 (90.33)	-85.2 ± 171.87	0.114	740.16 (462.02)	591.59 (318.76)	-125.4 ± 224.52	0.064	0.352
Sodium (mg)	1691.38 (1277.78)	1410.86 (1062.34)	-168.7 ± 441.90	0.241	1901.42 (1490.58)	1262.56 (615.57)	-679.4 ± 544.81	0.002*	0.385
Potassium (mg)	2109.42 (1143.25)	1612.23 (357.89)	-663.9 ± 596.31	0.013*	2326.14 (1733.65)	1835.24 (648.25)	-526.5 ± 700.02	0.023*	0.047*
Magnesium (mg)	215.75 (114.67)	198.19 (50.36)	-49.9 ± 74.62	0.074	285.05 (218.69)	187.69 (88.16)	-87.2 ± 89.09	0.007*	0.710
Phosphorus (mg)	956.06 (370.23)	850.60 (103.51)	-183.5 ± 232.59	0.047*	1202.46 (653.61)	854.51 (350.66)	-312.2 ± 356.88	0.009*	0.951
Iron (mg)	8.87 (4.75)	7.50 (2.37)	-1.7 ± 2.82	0.093	12.15 (7.78)	7.24 (2.86)	-4.8 ± 3.74	0.004*	0.620
Zinc (mg)	9.06 (4.01)	7.54 (1.02)	-2.0 ± 2.29	0.017*	11.79 (3.42)	7.13 (1.52)	-3.6 ± 3.05	0.002*	0.710
Caffeine (mg)	50.00 (27.25)	50.00 (25.00)	-1.9 ± 9.27	0.546	40.00 (19.00)	36.00 (41.50)	-1.5 ± 15.10	0.572	0.181

Median with interquartile range, ^a^ Mann-Withney U test, other tests Wilcoxon test, *p < 0.05

The comparison of diet qualities is given in Table 5. A statistically significant decrease was found in the mean sodium score of HEI-2015 components in the TRE group (p < 0.05). In the ERD group, the HEI-2015 total score and HEI-2015 components of total fruits, whole fruits, total vegetables, whole grains, dairy, added sugars, and saturated fats were statistically significant increase was determined (p < 0.05). There was a statistically significant difference between the groups in the mean scores of HEI-2015 total score and HEI-2015 components of total fruit total fruits, whole fruits, total vegetables and whole grains (p < 0.05). While 69.2% of the individuals had poor diet quality before the study, there were no individuals with poor diet quality after the intervention in the ERD group (p < 0.05).

**Table 5:** Comparison of the diet quality of the individuals

HEI-2015 Components	Maximum HEI-2015 Score	TRE group (n = 10)			ERD group (n = 13)			Statistical analysis of during the study between the two groups^c^
		Before study Mean (SD)	During study Mean (SD)	p-value	Before study Mean (SD)	During study Mean (SD)	p-value	
Total Fruits	5	1.32 (1.46)	1.21 (1.06)	0.878	0.79 (0.86)	2.99 (1.61)	0.001*	0.009*
Whole Fruits	5	2.17 (1.99)	2.29 (1.89)	0.721	1.37 (1.60)	4.09 (1.33)	0.002*	0.011*
Total Vegetables	5	2.83 (1.11)	1.85 (0.59)	0.139	1.93 (0.94)	2.94 (1.21)	0.033*	0.018*
Greens and Beans	5	1.83 (2.04)	2.34 (1.41)	0.646	2.07 (1.20)	2.99 (1.70)	0.133	0.264
Whole Grains	10	0.25 (0.80)	1.22 (3.12)	0.465	1.34 (2.84)	4.66 (3.80)	0.011*	0.041*
Dairy	10	1.60 (0.87)	1.97 (0.85)	0.646	1.49 (1.39)	3.26 (2.03)	0.023*	0.154
Total Protein Foods	5	4.73 (0.52)	4.54 (0.88)	0.500	4.44 (1.00)	4.89 (0.37)	0.128	0.159
Seafood and Plant Proteins	5	4.97 (0.08)	4.88 (0.35)	0.655	4.88 (0.39)	5.0 (0.0)	0.317	0.254
Fatty Acids	10	4.96 (2.92)	4.84 (3.21)	0.594	3.72 (2.47)	4.56 (2.82)	0.345	0.828
Refined Grains	10	6.14 (3.00)	3.24 (4.11)	0.074	6.01 (2.79)	5.62 (3.16)	0.695	0.119
Sodium	10	9.08 (1.96)	7.45 (3.53)	0.043*	9.09 (2.03)	8.71 (2.39)	0.374	0.373
Added Sugars	10	8.71 (3.01)	8.69 (2.80)	0.071	6.12 (4.31)	9.89 (0.37)	0.006*	0.340
Saturated Fats	10	3.32 (2.22)	4.56 (2.53)	0.333	2.73 (2.94)	5.27 (2.72)	0.039*	0.535
Total diet quality	100	51.9 (9.5)	49.1 (16.5)	0.508	46.1 (11.7)	65.0 (9.5)	0.001*	0.018*
*Poor diet*		4 (%40)	6 (%60)	0.500^a^	9 (%69.2)	-	0.021* ^**a**^	0.005* ^**b**^
*Needs improvement*		6 (%60)	4 (%40)		4 (%30.8)	12 (%92.3)		
*Good diet*		-	-		-	1(%7.7)		

^a^McNemar-Bowker test, ^b^Chi-square test, ^c^Mann-Withney U test, other tests Wilcoxon test, *p < 0.05

The consumption amounts of the food groups calculated from the food consumption record of the individuals in the TRE group are given in Fig. 1A. It was determined that there was a statistically significant decrease in the consumption of red meats, vegetables, solid fats and oils (p < 0.05).

**Fig. 1A:**
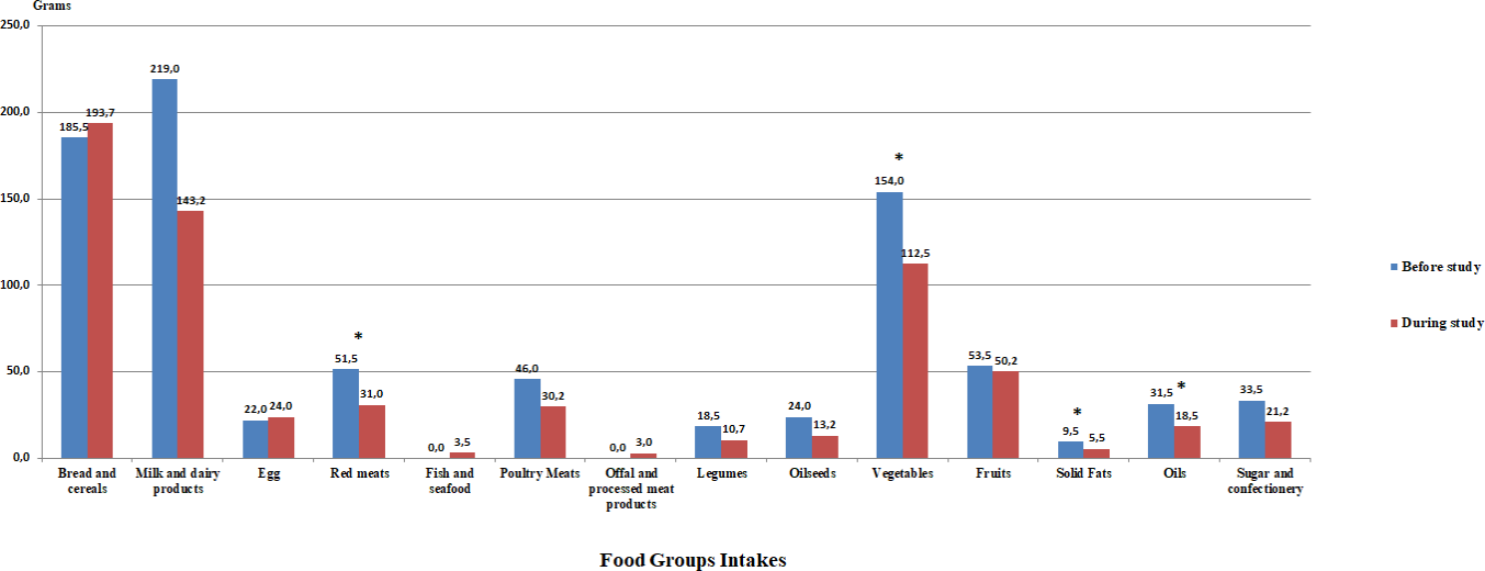
Consumption of Food Groups in the Time-Restricted Eating Group Wilcoxon test, *p < 0.05

The consumption amounts of the food groups calculated from the food consumption record of the individuals in the ERD group are given in Fig. 1B. A statistically significant decrease was determined in the consumption of bread and cereals, oilseeds, solid fats, oils, sugar and confectionery. It was determined that there was a significant increase in the amount of fruits consumption (p < 0.05).

**Fig. 1B:**
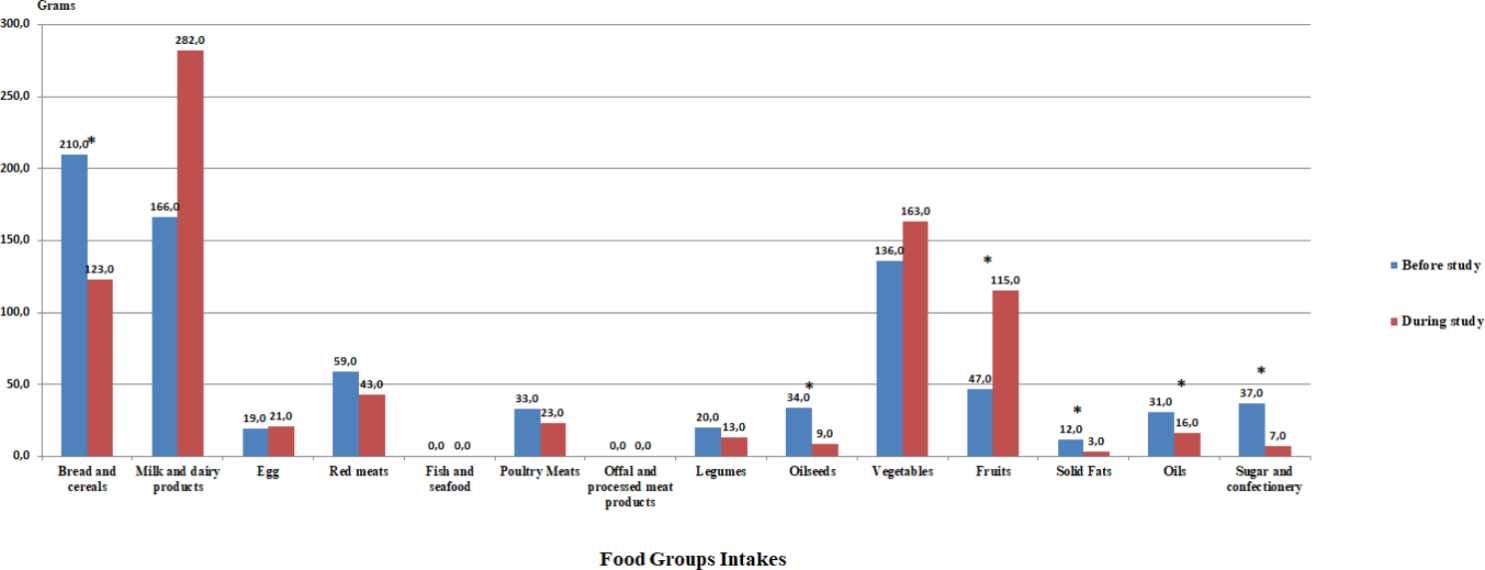
Consumption of Food Groups in the Energy-Restricted Diet Group Wilcoxon test, *p < 0.05

Comparison of the consumption amounts of the food groups during the study of the individuals in the TRE and ERD groups is given in Fig. 1C. While the amount of sugar and confectionery consumption was significantly higher in the TRE group; it was determined that the consumption amount of bread and cereals, milk and dairy products, vegetables and fruits was significantly higher in the ERD group (p < 0.05).

**Fig. 1C:**
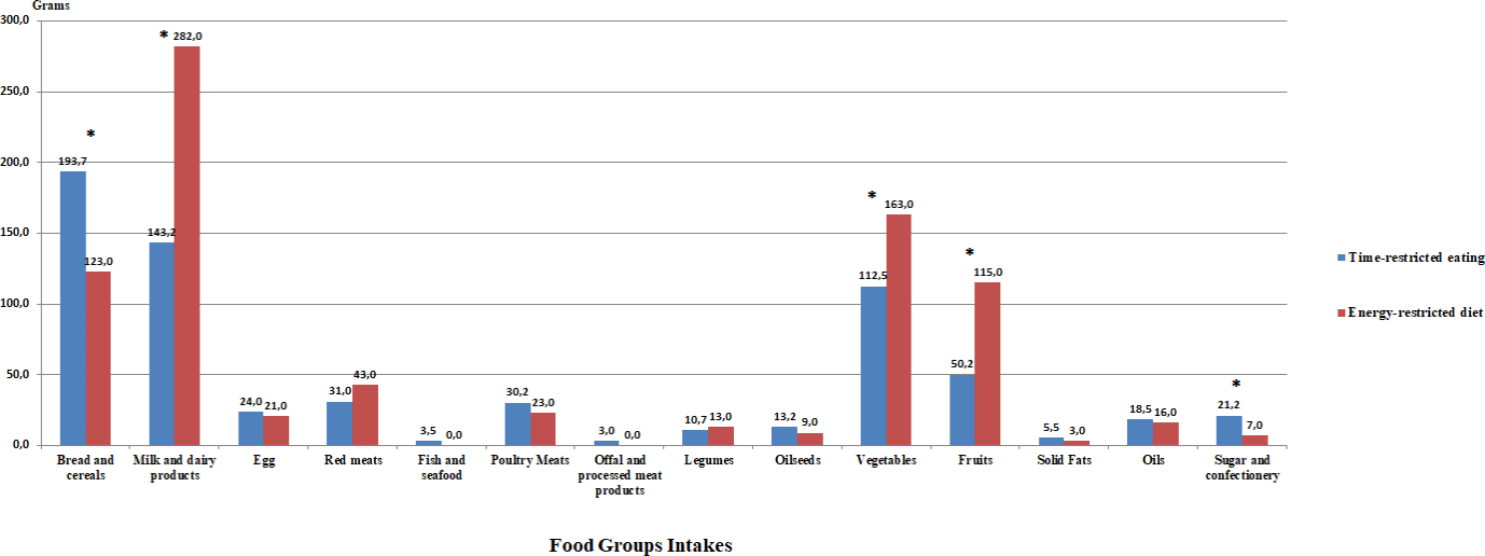
Comparison of Consumption of Food Groups Between Groups Mann-Withney U test, *p < 0.05

## Discussion

In this study, TRE and ERD were compared in terms of nutritional status and diet quality. According to the results of the study, body weight loss was observed and some biochemical parameters were improved in both ERD and TRE. However, only the individuals in the ERD group had an increase in diet quality and a decrease in body fat percentage.

Time-restricted eating is promising in terms of body weight loss in this study. It was determined that the overweight individuals in the TRE group had a 3.2% decrease in body weight, but no change was found in body fat percentages. Consistent with this study, Chow et al. [42] applied a limited diet of 8 h for 12 weeks and, while a 3.7% decrease in body weight of individuals was observed, no change was found in body fat percentages. In previous similar studies, body weight loss was observed in individuals [43–46], but contrary to this study, most of studies conducted showed a significant decrease in body fat percentages with loss of body weight in individuals [43, 45, 47, 48]. On the other hand, in this study, significant reductions in waist circumference, waist/height ratio, BMI, visceral fat and abdominal fat are very important in terms of preventing obesity and related diseases, and the results suggest that TRE may be an effective dietary intervention for noncommunicable diseases. Similar to this study, in different studies, a significant decrease was found in the waist circumference, and it was concluded that TRE has the potential to reduce the risk of abdominal obesity and cardiometabolic disease [45, 49].

In all of the studies within our knowledge in the literature, it has been shown that the ERD has positive effects on anthropometric measurements and body composition in parallel with this study [50–53]. In this study, individuals’ body weight decreased by 5.5% and body fat percentage by 2.7%. Also, it was determined that the decrease in waist circumference, waist/height ratio and BMI. In the study of Catenacci et al. [51], 12 obese individuals consumed 400 kcal of diet deficient in energy requirement for 8 weeks. 6.2% decrease in body weight and 1.0% decrease in body fat percentage of individuals were determined. In the study of Carter et al. [54], 24 obese individuals consumed a diet containing 1200–1500 kcal of energy for 12 weeks. 5.6% reduction in body weight and a 2.1% reduction in body fat percentage were observed. In the study of Varady et al. [55], 12 obese individuals followed a 25% ERD for 12 weeks, and a 5.0 ± 1.4% decrease in body weight was found. Changes in body weight and composition of individuals may differ depending on the methodology and duration of the studies.

In this study, a decrease was observed in the REE of individuals in both groups, but these changes were not statistically significant. In addition, individuals maintained their physical activity levels during the study, therefore, no significant change was determined in the TEE of the individuals. Similar to this study, it was concluded that TRE [47, 56] and ERD practices [53] did not provide a significant change in REE of individuals.

Eight weeks after the intervention, a significant decrease was found in total cholesterol and LDL-C in the TRE group. In a study conducted by Wilkinson et al. [45] parallel to this study, a significant decrease was determined in total cholesterol (7%) and LDL-C (11%). The results of this study support the improvement in the lipid profile in the fasting state. The results obtained were very important in terms of reducing the risk of cardiovascular disease and improving metabolic health. The proposed mechanism in this regard is the changes in the expression of peroxisome proliferator activated receptor alpha (PPAR-α) and peroxisome proliferator activated receptor gamma coactivator 1alpha (PGC-1α) in the liver during fasting. According to this mechanism; expression of PPAR-α and PGC-1α leads to increased fatty acid oxidation and apolipoprotein A synthesis, and decreased apolipoprotein B synthesis. Fatty acid oxidation causes a decrease in liver triglyceride level and very low density lipoprotein production. Serum cholesterol and triglyceride concentrations decrease. Fasting practices can reduce serum LDL-C levels by decreasing liver apolipoprotein B synthesis [57].

There was a significant decrease in total cholesterol levels of individuals in the ERD group. In the study of Catenacci et al. [51] 12 individuals consumed 400 kcal of diet deficient in energy requirement. At the end of eight weeks, a decrease in the total cholesterol was detected. In the study of Molina-Jiménez et al. [58], it was determined that an ERD had a positive effect on the total cholesterol level of individuals. Healthy nutrition recommendations are given to individuals with an ERD. In this study, it was determined that there was a significant decrease in the intakes of fat, saturated fat and cholesterol (p < 0.05). It is thought that the change in food consumption of individuals has a positive effect on the total cholesterol level.

It is known that adipokine levels change depending on body weight loss. Circulating concentrations of adipokines produced by adipocyte (excluding adiponectin) tend to decrease with body weight loss, while adiponectin concentration tends to increase [59–61]. In a study, after 60% energy restriction, an increase in adiponectin levels and a 15% decrease in body weights were found in 35 obese individuals at the end of 8 weeks [62]. In the study of Christiansen et al. [63], obese individuals followed an energy restriction of 55% and 65% for 12 weeks. 8% and 11% decrease in body weights of individuals, respectively, and a significant increase in adiponectin levels were detected. The results of the studies are similar to this study. In this study, individuals in the ERD group had a decrease in body weight, while an increase was observed in adiponectin levels. Adiponectin is anti-atherogenic and has an insulin-sensitizing effect [64, 65]. It can be said that the increase in the adiponectin levels of the individuals in this study may cause positive effects in terms of health.

The antioxidant defense system plays a role against reactive oxygen species and oxidative stress in the body. Antioxidants prevent or reduce the damage caused by oxidation in body tissues by scavenging free radicals, reactive oxygen and nitrogen species. The main source of non-enzymatic antioxidants is a balanced diet. The main nutrients that are sources of antioxidants in the diet are vegetables and fruits containing vitamins A, E and C [66, 67]. In this study, a significant increase was found in the TAS who followed an ERD for 8 weeks (p < 0.05). Parallel to this finding, there was a slight increase in the consumption of vegetables and a significant increase in the consumption of fruits. (p < 0.05). Again, it was determined that there was a significant increase in total fruits and total vegetables, which are components of diet quality (p < 0.05).

In this study, it was determined that the mean energy intake of individuals in the TRE group decreased by 405.7 ± 448.60 kcal. No change was found in the percentages of energy from macronutrients. In a study, 23 obese individuals fasted for 16 h, and a decrease of 341 ± 53 kcal/day was found in the energy intake of individuals [46]. In the study of Cienfuegos et al. [44], 35 obese individuals applied limited eating for 4 and 6 h. It was observed that the energy intake of individuals in both groups decreased by an average of 550 kcal/day [44]. In a study in which individuals fasted for 11 h, a decrease of 240 kcal/day was found in their energy intake [68]. Similar to this study, a decrease in energy intake was found in studies and the reductions in energy intake vary in relation to the fasting periods applied by the individuals. Also, parallel to this study, no significant change was observed in the percentages of energy from macronutrients in the previous studies [46, 47, 68]. This result is thought to be due to the lack of any intervention in the food intake of the individuals. Depending on the decrease in energy intake a significant decrease was observed in certain nutrients in both ERD and TRE groups. In the study of Conley et al. [52] obese individuals consumed energy restricted diet, and in parallel with this study, a significant decrease was found in macro and micronutrient values due to the decrease in energy intake of individuals.

This is one of the limited number of studies investigating the effect of TRE on diet quality. In this study, it was determined that TRE did not have a significant effect on diet quality. However, none of the individuals in this group had good diet quality at the beginning of the study. The mean HEI score of individuals is 49.1 and dietary intervention is required in these individuals. Time-restricted eating is a dietary approach that does not interfere with the diet applied, only the time of food consumption is limited [12, 13]. In parallel with this study, it was determined in previous studies that TRE did not have any effect on diet quality [23, 24]. However, while talking about the possible positive effects of fasting on metabolism, the effects of long-term fasting on diet quality should not be ignored.

In this study, it was determined that there was a significant decrease in the consumption amounts in the bread and cereals, oilseeds, solid fats and oils, sugar and confectionery and a significant increase in the fruits in the ERD group (p < 0.05). Compared with the pre-study, a significant increase was found in the HEI components of total fruits, whole fruits, total vegetables, whole grains, dairy, added sugars, saturated fats, and HEI total diet quality score (p < 0.05). While 69.2% of the individuals had poor diet quality before the study, there was no individual with poor diet quality in this group with the consumption of ERD. Energy-restricted diet provided a positive change in the diet quality of the individuals. Similar results have been demonstrated by Sundfor et al. [69], Bracci et al. [70] and Ptomey et al. [71].

This study had some limitations. The sample of this study was limited. The effect of these dietary practices on biochemical parameters, body composition, and diet quality should be extensively investigated with further studies with larger sample sizes. Planning and conducting studies with longer follow-ups and high sample sizes are very important in terms of creating public health-based recommendations.

## Conclusion

In the light of current data, TRE emerged as an alternative method of energy restriction and body weight loss. However, no change was detected in the body fat percentage of the individuals. The decrease in blood cholesterol levels (total cholesterol and LDL-C) in the TRE group was was very important. Energy-restricted diet had a positive effect on individuals’ body weight, body fat percentage and body composition. Also, the TRE has improved total cholesterol, total antioxidant status and adiponectin levels. While ERD affected the diet quality of individuals positively, TRE did not have any effect on diet quality. The effects of TRE on diet quality and diet pattern should be evaluated with further studies in terms of long-term health outcomes.

## Data Availability

The datasets generated and/or analysed during the current study are not publicly available due to privacy or ethical restrictions, but are available from the corresponding author on reasonable request.

## List of Abbreviations

BMI: Body mass index
ERD: Energy-restricted diet
HDL-C: High density lipoprotein cholesterol
HEI: Healthy eating index
HOMA-IR: Homeostatic model assessment of insulin resistance
IL-6: Interleukin 6
LDL-C: Low density lipoprotein cholesterol
OSI: Oxidative stress index
PAL: Physical activity level
PAR: Physical activity ratio
PGC-1α: Peroxisome proliferator activated receptor gamma coactivator 1alpha
PPAR-α: Peroxisome proliferator activated receptor alpha
REE: Resting energy expenditure
TAS: Total antioxidant status
TEE: Total energy expenditure
TOS: Total oxidant status
TRE: Time-restricted eating
